# Constipation

**Published:** 1884-10

**Authors:** 


### CONSTIPATION.

THERE is a vast deal of truth concentrated in the poet's statement that "There's a poison drop in man's purest cup." Unalloyed pleasure has not yet been vouchsafed to man. This fact, so patent on all sides, is not due to any deliberate design on the part of a beneficent Creator, but rather to the failure on the part of man, to fulfil the conditions of perfect beatitude. This failure is either the result of deliberate contravention of nature's laws, or is due to an ignorance of these laws. When men shall have learnt to thoroughly understand these laws, and to yield unreserved obedience thereto, their happiness will, doubtless, be complete. Such knowledge and such obedience, however, imply a state of perfection which is in the very remote future.

Referring to the drawbacks to complete happiness under the civilization, enlightenment and far-pervading wealth of the present age, we find them to exist principally in the discomforts and diseases arising from an improper cuisine and deficiency of exercise. Among the discomforts or diseases thus arising, to which we would now more particularly refer, is constipation. The prevalence of this condition detracts from the sum of happiness to an extent which cannot be appreciated except by one competent to discriminate the train of complex ills directly traceable to it. The intestines may aptly be called the main sewer of the body. While very important products of waste are eliminated through other channels, this is that through which the great bulk is carried off. Physical well-being depends quite as much—and indeed more—on a proper discharge of waste than on a proper supply of food. The body generates poisons which, if they were not carried off, would be speedily fatal; and to the extent that they are retained, through arrest of the function of the intestines and other emunctories, we have physical discomfort and disease. A surprising percentage of such discomfort and disease is traceable to constipation This fact is very generally recognized, and as a consequence, we have the immense popularity and use of cathartics and nostrums whose chief virtue lies in their cathartic properties. The great defect in the cathartics in vogue, lies in the fact that they work only temporary relief, and instead of improving the condition to which the constipation is due, they but aggravate it.

Constipation is due to deficient tone or contractile power in the muscular coats of the intestines. While a brisk cathartic of Epsom salts, for instance, may unload an accumulation which the diminished contractile power of the intestines is incompetent to propel onward, it does so by stimulating the muscular fibre. Now, it is a philosophical law that stimulation is followed by depression, and this law is illustrated in the increased torpidity of the bowels and constipation which follows a brisk catharsis What is required in the treatment of constipation is a remedy which, while evacuating the intestines, restores the lost tonicity of the muscular coats to which the difficulty is due. Physicians seek to effect this double object by combining such drugs as nux vomica or strychnia, etc., with the laxatives which they prescribe. Drugs of the latter nature must be employed with great circumspection, and never without the advice and under the supervision of the physician. They are thus not adapted to domestic use.

A harmless tonic cathartic that seems to have grown into great favor during the past few years, is Rhammer's purshiana, or Cascara Sangrada. This drug is the bark of a small tree found on the Pacific coast in this country; the excessive bitterness of the crude bark has been overcome by the use of aromatics in the preparation known as Cascara cordial. It operates without griping, and has the great advantage of imparting tone to the muscular fibre of the intestines.

The mistake is often made by the patient of "pinning his faith" to a single remedy, and hence he is often disappointed in the result. In addition to the use of cascara cordial, he should continue the employment of those means which all have found advantageous : as bread made from whole wheat flour, pearled oats, etc., besides the free use of fresh fruits. We do not, as a rule, recommend the use of enemas; but there are cases of constipation that bid defiance to all other remedies, and in these cases they should be resorted to, cautiously but persistently, until the object is accomplished; their daily use may become imperative during life. We have frequently recommended the more simple, and usually more effective plan of using suppositories conjointly with the use of cascara cordial, as above referred to.

When it is considered that habitual constipation is the cause of more than half of the diseases that afflict mankind, and that it cannot exist for a long time without undermining the constitution, no thoughtful person will neglect the employment of any safe remedies that afford the remotest promise of relief.

---

Nobody can tell how many disputes for the front side of the bed have been settled by moving the bedstead into the centre of the room.

